# Spatiotemporal analysis of schistosomiasis and soil-transmitted helminth distribution in three highly endemic provinces in Angola

**DOI:** 10.1371/journal.pntd.0012974

**Published:** 2025-04-08

**Authors:** Adam W. Bartlett, Tatiana Proboste, Elsa P. Mendes, Marta S. Palmeirim, Ana Direito, Ricardo J. Soares Magalhaes, Sergio Lopes, Susana Vaz Nery

**Affiliations:** 1 Neglected Tropical Diseases Research Group, Global Health Program, Kirby Institute, University of New South Wales, Sydney, Australia; 2 UQ Spatial Epidemiology Laboratory, School of Veterinary Science, University of Queensland, Gatton, Australia; 3 Queensland Alliance for One Health Sciences, School of Veterinary Science, University of Queensland, Gatton, Australia; 4 National Directorate of Public Health, Ministry of Health, Luanda, Angola; 5 The MENTOR Initiative, Huambo, Angola; 6 Children’s Health and Research Centre, Children’s Health and Environment Program, University of Queensland, South Brisbane, Australia; Rio de Janeiro State University: Universidade do Estado do Rio de Janeiro, BRAZIL

## Abstract

**Background:**

A school-based preventive chemotherapy (PC) program has operated since 2013 for soil-transmitted helminths (STHs) and 2014 for schistosomiasis in Huambo, Uige and Zaire provinces, Angola. This program was informed by a prevalence survey in 2014 and evaluated in 2021, demonstrating limited impact in reducing provincial-level prevalence. This geospatial analysis aims to provide granular estimates of the geographic distribution of schistosomiasis and STHs to target control strategies.

**Methods:**

Parasitological data on schistosomiasis and STHs were obtained from school-based prevalence surveys conducted in 2014 and 2021. These data were supplemented with open access environmental and climatic data to develop risk prediction maps for each parasite at each time point. Variables for the final risk prediction models were selected through non-spatial multivariable regression analyses and residual spatial autocorrelation was investigated using semivariograms. Risk prediction maps were then developed using either non-spatial or spatial (using the Matérn covariance) geostatistical models depending on the presence of residual spatial autocorrelation.

**Results:**

The 2014 survey included 17,093 schoolchildren (575 schools) for schistosomiasis and 3,649 schoolchildren (121 schools) for STHs, and **t**he 2021 survey included 17,880 schoolchildren (599 schools) for schistosomiasis and 6,461 schoolchildren (214 schools) for STHs. Our analyses indicated that in Huambo province, there are small areas of high schistosomiasis risk in the north and south, and a centrally located STH hotspot, with no discernible change in predicted risk for either infection over time. In Uige, there has been a reduction in schistosomiasis hotspots in the southwest corner but an overall increase in predicted risk throughout the province, whilst there is a concerning trend for expanding areas of high predicted STH risk throughout. In Zaire, there are increasing areas of higher risk for schistosomiasis and STHs, with co-endemic hotspots.

**Conclusion:**

These risk prediction maps importantly identify higher risk areas for schistosomiasis and STHs within large geographic regions that should be prioritised for control with tailored decisions for future PC delivery.

## Author summary

To combat soil-transmitted helminth (STH) infections and schistosomiasis a school-based preventive chemotherapy program has been operating since 2013 and 2014, respectively, in the Angolan provinces of Huambo, Uige and Zaire. The program was informed by a prevalence survey conducted in 2014 followed by an impact assessment in 2021. This analysis sought to develop spatial risk predictions of STHs and schistosomiasis using data collected from the 2014 and 2021 surveys, in conjunction with environmental and climatic factors, to improve understanding of how the epidemiology of STHs and schistosomiasis has changed in the context of the program. Our study shows that there have been slight reductions in higher-risk areas for schistosomiasis and STH infections in Huambo. However, there is a concerning trend for expanding areas of high STH infection risk throughout Uige province and the evolution of co-endemic schistosomiasis and STH infection hotspots in Zaire that should be prioritized for targeted interventions.

## Introduction

Schistosomiasis and soil-transmitted helminth (STH) infections are two of the major contributors to the global burden of neglected tropical diseases (NTDs) [1], and have been targeted by the World Health Organization (WHO) for their elimination as a public health problem by 2030 [2]. Integral to the understanding of schistosomiasis and STH epidemiology and tracking progress towards the WHO 2030 targets is the conduct of parasitological surveys to estimate the prevalence and burden, as measured by infection intensity, of these infections. The conventional design for schistosomiasis and STH surveys are cluster surveys involving the selection of a subset of schools and schoolchildren to provide samples for analysis [3]. The results from these school cluster surveys are then used to generate an estimate of the prevalence and burden of schistosomiasis and STHs in their respective administrative unit (e.g., municipality, commune) that informs the implementation of control strategies [3]. A limitation of these conventional methods is the lack of granularity in identifying different areas of risk within a given municipality or district or areas that cross administrative boundaries [4]. To overcome these limitations, environmental and climatic variables that influence transmission of the infectious agents can be used to develop risk prediction models for infection [5,6] that are able to predict risk of infection across a given geographic area based on cluster level infection data. Furthermore, the incorporation of environmental and climatic variables importantly accounts for schistosomiasis and STH transmission dynamics related to environmental factors and climatic conditions for the at-risk human population as well as the vectors and animal reservoirs of the infectious agents [7–12].

As part of national efforts to control schistosomiasis and STHs, baseline school cluster surveys were performed in Angola in three provinces in 2014 [13] and the 15 remaining provinces in 2018-19 [14]. The 2014 survey identified ecological zone as a risk factor for both schistosomiasis and STHs [13], and both surveys demonstrated considerable geographical variability in schistosomiasis and STH prevalence [13,14]. These surveys informed the implementation of a school-based preventive chemotherapy (PC) program, for which an impact assessment was subsequently undertaken in the first three provinces in 2021 [15]. This demonstrated a limited impact of the school-based PC program in reducing the prevalence of schistosomiasis and STHs across the provinces of Huambo, Uige and Zaire [15].

Given the limited impact of the schistosomiasis and STH control program to date, we sought to develop spatial risk predictions using data collected from the 2014 and 2021 schistosomiasis and STH surveys to improve understanding of the epidemiology of schistosomiasis and STH across Huambo, Uige and Zaire provinces in Angola and how this has changed in the context of a school-based PC program. This will provide estimates of the geographical distribution of schistosomiasis and STHs at a smaller spatial scale to better target control strategies.

## Methods

### Ethics statement

Ethics approval was obtained from the Ministry of Public Health of Angola (101/GD/DNSP/2014 and 17/C.E./2021), and from the University of New South Wales, Sydney Australia (reference HC210192). For the primary parasitological data collection, informed written consent was obtained from the school directors of each school to allow field teams to visit. Parents/guardians at the school were then provided all the relevant study information and informed written consent was obtained by parents/guardians of schoolchildren present on the day of field teams visiting to participate in the surveys.

### Study area and data collection

**Fig 1:**
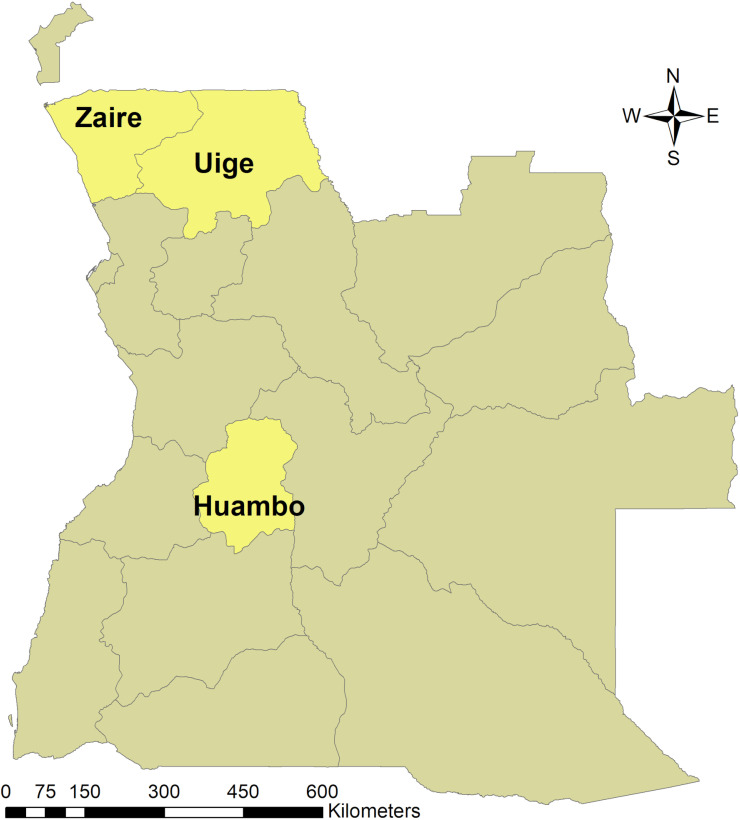
Location of Huambo, Uige and Zaire provinces in Angola. Base-layer map provided by the Database of Global Administrative Areas (GADM): https://gadm.org/download_country.html; license: https://gadm.org/license.html.

Angola is situated on the west coast of sub-Saharan Africa (Fig 1), with an overall area of 1,246,700km^2^ that comprises six homogenous ecological zones [16]. The country consists of 18 provinces and 164 municipalities [16], with municipalities considered the conventional administrative unit for the implementation of NTD control programs in Angola.

The schistosomiasis and STH school-based cluster surveys were designed to estimate the prevalence of these infections at the province level with representation from each municipality across Huambo, Uige and Zaire provinces. Data was collected through field teams visiting selected schools in each municipality and obtaining and analysing urine and stool samples from a subset of schoolchildren. Full details of the school and student selection for each of the surveys are reported elsewhere [13,15].

The urine rapid diagnostic tests (RDTs) of circulating cathodic antigen (CCA) (Rapid Medical Diagnostics, Pretoria, RSA) and Hemastix (Bayer UK), to detect haematuria as a proxy for infection, were used to detect *Schistosoma mansoni* and *Schistosoma haematobium* infections, respectively. Trace readings for both RDTs were considered indicative of infection in both surveys. The Kato-Katz microscopy technique was used to detect and measure intensity of infection for STH species (*Ascaris lumbricoides*, hookworm, and *Trichuris trichiura*) in stool samples. Parasitological data was entered manually into data entry forms by field workers, which was then collated and entered in the Expanded Special Project for the Elimination of Neglected Tropical Diseases (ESPEN) Collect Tool (2014 and 2021 surveys).

### Obtaining and processing environmental and climatic data

Contemporary environmental and climatic data (Table 1) were collected for each of the parasitological surveys, including: temperature, precipitation, elevation, vegetation, landcover soil pH (for STH analysis), and distance to water bodies (for schistosomiasis analysis). Extraction for temperature and precipitation data was performed using the ‘raster’ R package [17]; elevation and soil data were extracted using the ‘geodata’ R package [18]; extraction of vegetation data was performed using the ‘MODIStsp’ R package [19]; and determining distance to water bodies using the ‘terra’ R package [20].

**Table 1:** Environmental data sources incorporated in the analysis.

Data type	Source	Temporal resolution	Spatial resolution (m)
Land surface temperature	WorldClim [21]	Monthly average 1970–2020	1000
Precipitation	WorldClim [21]	Monthly average 1970–2020	1000
Elevation	Amazon Web Services Terrain Tiles [22]		30
Vegetation (NDVI, EVI)	MODIS Terra satellite [19]	Mar-Apr 2014^a^ May-Aug 2021^b^	1000
Landcover	MODIS Terra and Aqua satellite [19]	Jan-Dec 2014^a^ Jan-Dec 2020^b^	500
Soil pH	International Soil Reference Information Centre [18]	2020^c^	250
Distance to water bodies	R package ‘terra’ [20]		1

^a^For 2014 parasitological survey. ^b^For 2021 parasitological survey. ^c^Soil data from the latest SoilGrids release (2020) was used for both surveys due to improved computational infrastructure and data inputs since the previous release (2017). MODIS = Moderate Resolution Imaging Spectroradiometer. NDVI = normalized difference vegetation index. EVI = enhanced vegetation index.

### Statistical analysis

To identify environmental and climatic variables for the schistosomiasis and STH infection risk prediction models, univariate generalized linear regression models (binomial family) were used to evaluate associations between school-level prevalence of schistosomiasis and STHs, for the 2014 and 2021 surveys, and each of the environmental and climatic variables (Table 1) separately. Variables with a p-value <0.2 were then assessed for collinearity, designated by a Pearson Correlation coefficient >0.9. Collinear variables with the highest Akaike Information Criterion (AIC) were removed from further multivariable analysis.

Multivariable generalized linear non-spatial regression models (binomial family) were subsequently built retaining variables with a p-value <0.05. Resultant multivariable models were then assessed for the presence of multi-collinearity within the set of predictors using the variance inflation factor (VIF), with a VIF<10 considered acceptable. The multivariable model with the lowest AIC that had all variables with a p-value <0.05 and a VIF<10 was chosen as the final model. Residual spatial autocorrelation for the regression models were then assessed using semivariograms. Models demonstrating residual spatial autocorrelation were then refitted with the Matérn covariance, using the ‘spaMM’ R package [23], to control for spatial effects.

Risk prediction maps were then developed for each of the three provinces from the final models for schistosomiasis and STH risk for each of the 2014 and 2021 surveys. Risk stratification was based on WHO guidelines that use prevalence as the key indicator [3]. For schistosomiasis, areas with a prevalence (as determined by Kato Katz for *S. mansoni* and urine filtration for *S. haematoboium*) of ≥50% are considered high risk, areas with a prevalence 10% to <50% considered moderate risk, and areas with a prevalence of 1% to <10% considered low risk [3]. Rapid diagnostic tests (instead of traditional microscopy methods) are being increasingly used for schistosomiasis surveys, prompting the WHO to release guidelines indicating a *S. mansoni* prevalence of 30% and 75% by CCA equivalent to 10% and 50% by single-smear Kato Katz [24]. For STHs, areas with a prevalence of ≥50% are considered high risk, while areas with a prevalence of 20 to <50% considered low risk [3]. All statistical analyses were performed using R Statistical Software, version 4.3.2.

## Results

### Parasitological survey populations

**Fig 2:**
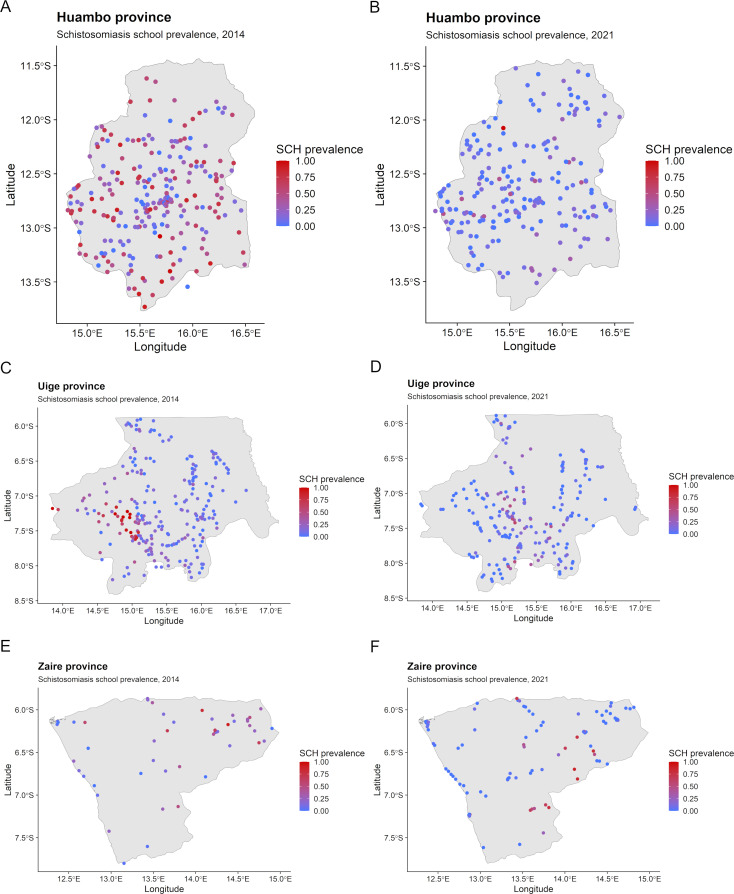
School-level prevalence for schistosomiasis (SCH) in 2014 and 2021 for (A–B) Huambo, (C–D) Uige and (E–F) Zaire provinces, Angola. Base-layer map provided by the Database of Global Administrative Areas (GADM): https://gadm.org/download_country.html;license: https://gadm.org/license.html.

**Fig 3:**
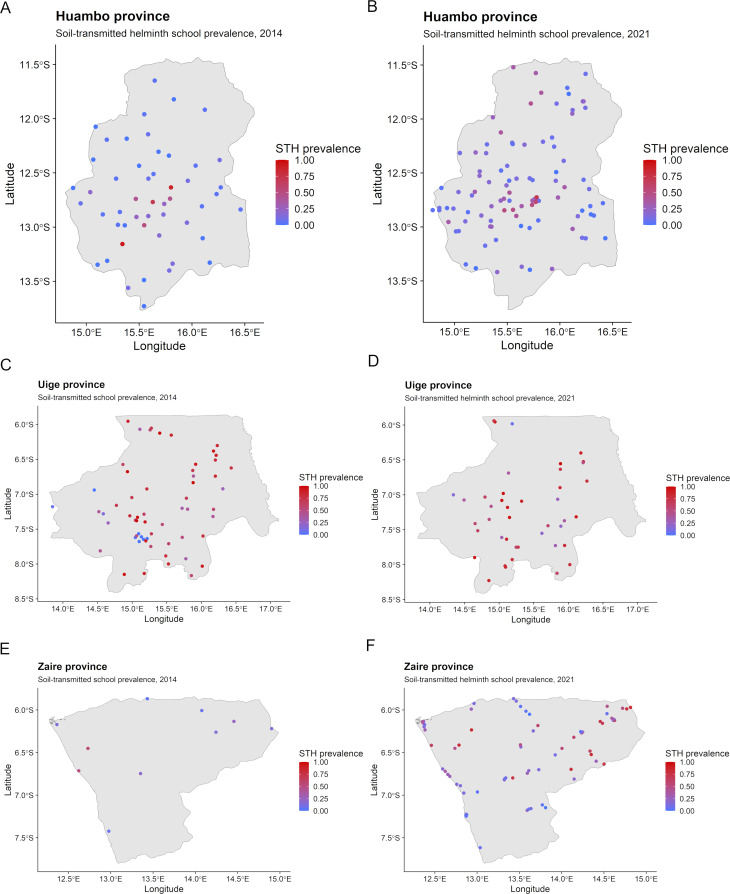
School-level prevalence for soil-transmitted helminths (STHs) in 2014 and 2021 for (A–B) Huambo, (C–D) Uige and (E–F) Zaire provinces, Angola. Base-layer map provided by the Database of Global Administrative Areas (GADM): https://gadm.org/download_country.html;license: https://gadm.org/license.html.

Parasitological data for the 2014 survey included 17,093 schoolchildren (from 575 schools) in the schistosomiasis survey and 3,649 schoolchildren (from 121 schools) in the STH survey. For the 2021 survey, 17,880 schoolchildren (from 599 schools) were included in the schistosomiasis survey and 6,461 schoolchildren (from 214 schools) were included in the STH survey. Study population and cluster-adjusted (at school-level) prevalence for schistosomiasis and STHs for the baseline survey and impact assessment are shown in Table 2 and have been published previously [13,15]. School-level prevalence from the baseline survey and impact assessment for schistosomiasis and STHs are visualized in Figs 2 and 3, respectively.

**Table 2:** Parasitological survey populations and cluster-adjusted province level schistosomiasis and soil-transmitted helminth prevalence.

	Schistosomiasis		Soil-transmitted helminths	
	**2014**	**2021**	**2014**	**2021**
**Total schoolchildren** **(schools)**	17,093 (575)	17,880 (599)	3,649 (121)	6,461 (214)
Prevalence* **(municipality range)**				
**Huambo**	34.7% (26.9-57.0%)	29.6% (20.7-39.7%)	13.1% (0.8-33.2%)	16.3% (4.1-29.2%)
**Uige**	25.3% (5.9-77.3%)	35.4% (17.0-53.9%)	49.4% (5.2-89.7%)	65.1% (26.7-95.6%)
**Zaire**	32.2% (6.9-51.2%)	28.3% (17.9-41.2%)	20.6% (6.7-36.8%)	28.2% (7.2-49.2%)

*Prevalence adjusted for clustering at the school level.

### Geospatial risk predictions for schistosomiasis

The variables used in the final multivariable non-spatial regression models for schistosomiasis in Huambo, Uige and Zaire provinces in 2014 and 2021 are shown in Table 3. For the 2014 risk predictions, a non-spatial model was used for Zaire as there was no residual spatial autocorrelation, while spatial models were used for Huambo and Uige due to residual spatial autocorrelation (see S1 Material for semivariograms). For the 2021 risk predictions, a non-spatial model was used for Huambo, while spatial models were used for Uige and Zaire (see S1 Material for semivariograms). The regression model co-efficients for the variables included in the final STH risk prediction models for 2014 and 2021 are shown in S2 Material.

**Table 3:** Variables included in the final risk prediction models for schistosomiasis risk in 2014 and 2021.

Year	Province	Variable	Odds Ratio	95%CI
**2014**	**Huambo**	Isothermality	1.06	1.01, 1.10
		Max temperature of warmest month	1.02	1.01, 1.04
		Annual precipitation	0.99	0.99, 1.00
		Precipitation of coldest quarter	1.03	1.01, 1.05
		*Non-spatial model: AIC = 3,338.6; Adjusted r-squared = 0.05*		
		*Spatial model: Residual variance (φ) = 0.003*		
	**Uige**	Mean temperature diurnal range	1.03	1.02, 1.04
		Temperature seasonality	1.00	1.00, 1.00
		Precipitation of coldest quarter	1.03	1.02, 1.03
		Elevation	1.00	1.00, 1.00
		NDVI	1.00	1.00, 1.00
		EVI	1.00	1.00, 1.00
		Distance to water bodies	1.00	1.00, 1.00
		*Non-spatial model: AIC = 1,787.1; Adjusted r-squared = 0.53*		
		*Spatial model: Residual variance (φ) = 0.02*		
	**Zaire**	Precipitation of Wettest Month	1.02	1.02, 1.03
		Precipitation of Driest Month	7.01	3.67, 13.33
		Distance to water bodies	1.00	1.00, 1.00
		*Non-spatial model: AIC = 569.57; Adjusted r-squared = 0.16*		
**2021**	**Huambo**	Annual mean temperature	1.03	1.02, 1.04
		Precipitation seasonality	0.95	0.92, 0.98
		*Non-spatial model: AIC = 1,888.3; Adjusted r-squared = 0.05*		
	**Uige**	Mean annual diurnal range	0.98	0.97, 0.99
		Temperature seasonality	1.00	1.00, 1.00
		Precipitation of wettest month	1.01	1.00, 1.01
		Elevation	1.00	1.00, 1.00
		Distance to water bodies	1.00	1.00, 1.00
		*Non-spatial model: AIC = 2,710; R-squared = 0.05*		
		*Spatial model: Residual variance (φ) = 0.01*		
	**Zaire**	Isothermality	1.31	1.23, 1.39
		Temperature seasonality	1.01	1.00, 1.01
		Precipitation of driest month	0.19	0.11, 0.31
		Precipitation seasonality	0.93	0.90, 0.96
		EVI	1.00	1.00, 1.00
		Landcover	0.78	0.66, 0.91
		*Non-spatial model: AIC = 695.11; R-squared = 0.35*		
		*Spatial model: Residual variance (φ) = 0.01*		

Odds ratio and 95%CI derived from non-spatial regression analysis. Metrics of model performance provided for non-spatial regression models and spatial regression models when used. Isothermality = (mean diurnal temperature range/ temperature annual range) x 100. AIC = Akaike information criterion. CI = confidence interval. EVI = enhanced vegetation index. NDVI = normalized difference vegetation index.

**Fig 4:**
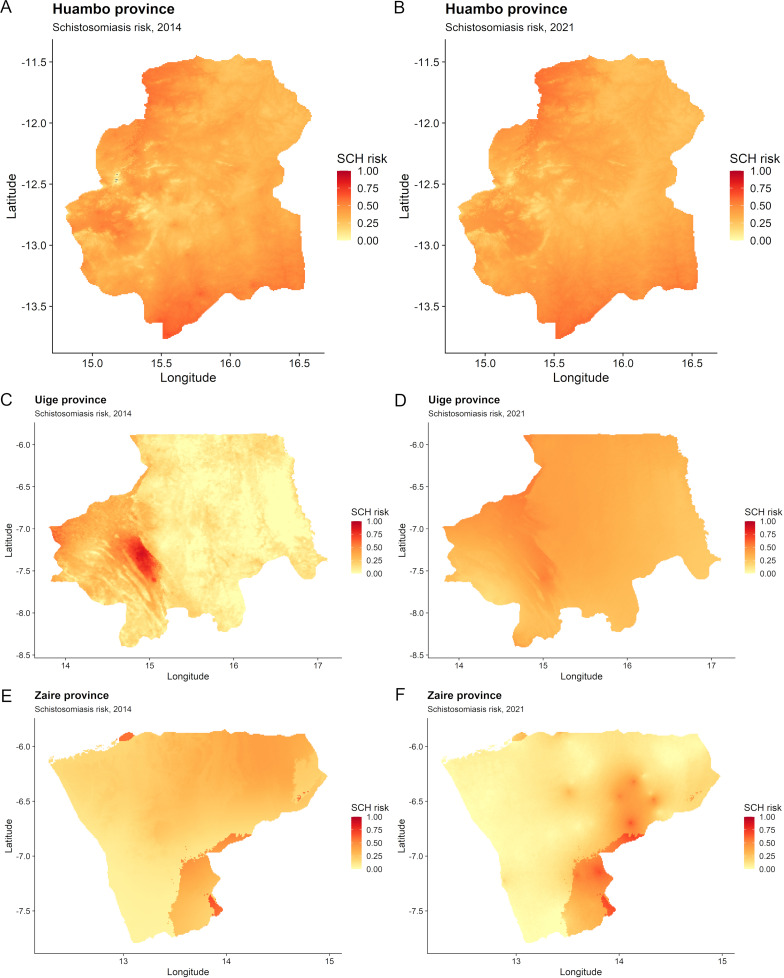
Risk prediction maps for schistosomiasis (SCH) in 2014 and 2021 for (A–B) Huambo, (C–D) Uige and (E–F) Zaire provinces, Angola. Low risk, <30%; Moderate risk, 30% to <75%; High risk, ≥75% (as determined by rapid diagnostic tests) (24). Base-layer map provided by the Database of Global Administrative Areas (GADM): https://gadm.org/download_country.html; license: https://gadm.org/license.html.

Our results of risk prediction for Huambo indicated that there are small areas of higher predicted risk in the northern and southern parts of the province, with no discernable change in predicted risk throughout the province between 2014 and 2021 (Fig 4). Most of the province is considered low to moderate risk for schistosomiasis in 2021 (predicted schistosomiasis prevalence < 30% and <75%, respectively, as determined by RDTs). In Uige, in 2014, the southwestern corner of the province had higher predicted risk (prevalence ≥30% by RDTs), with much of the remaining parts of the province low risk (prevalence <30% by RDTs). In 2021, there has been a reduction in hotspots throughout the southwest corner, however there is a general increase in predicted schistosomiasis risk throughout the eastern and central parts of Uige with a predicted risk of 30% to <75%. For Zaire, most of the province was found to be low risk for schistosomiasis (<30% predicted prevalence by RDTs) in 2014, however there has been demonstrable development of hotspots across north-eastern parts of the province with predicted schistosomiasis prevalence of greater than 75% by RDTs in 2021.

### Geospatial risk predictions for soil-transmitted helminths

**Fig 5:**
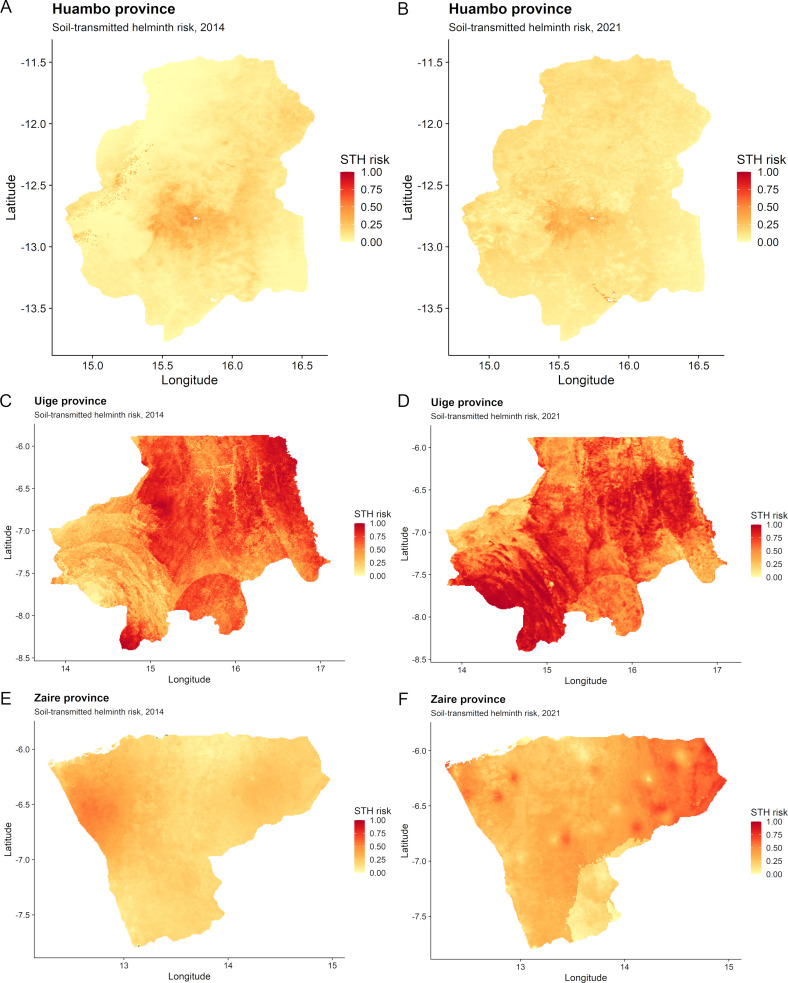
Risk prediction maps for soil-transmitted helminths (STHs) in 2014 and 2021 for (A–B) Huambo, (C–D) Uige and (E–F) Zaire provinces, Angola. Low risk, 20 to <50%; High risk, ≥50% (as determined by stool microscopy) (3). Base-layer map provided by the Database of Global Administrative Areas (GADM): https://gadm.org/download_country.html;license: https://gadm.org/license.html.

Table 4 shows the variables used in the final multivariable non-spatial regression models for STHs in Huambo, Uige and Zaire provinces in 2014 and 2021. For the 2014 risk predictions, spatial models were used for Huambo and Zaire due to residual spatial autocorrelation, but not for Uige (see S3 Material for semivariograms). For the 2021 risk predictions, non-spatial models were used for Huambo and Uige, while a spatial model was used for Zaire due to residual spatial autocorrelation (see S3 Material for semivariograms). The regression model co-efficients for the variables included in the final STH risk prediction models for 2014 and 2021 are shown in S4 Material. The resultant spatial risk prediction maps for STHs are presented in Fig 5. For Huambo, the STH hotspot (≥50% predicted prevalence) identified in the central region in 2014 has reduced in size in 2021. In Uige, most of the province is considered high risk for STHs (predicted prevalence ≥50%) in both 2014 and 2021, with a transition in the highest risk areas shifting from central and eastern regions towards south-western parts of the province over time. For Zaire, noticeable STH hotspots have developed throughout north and north-eastern parts of the province with a predicted STH prevalence of ≥50% in 2021. These predicted STH hotspots correspond overlap with predicted schistosomiasis hotspots (Fig 2), indicating co-endemic hotspots.

**Table 4:** Variables included in the final risk prediction models for soil-transmitted helminths risk in 2014 and 2021.

Year	Province	Variable	Odds Ratio	95%CI
**2014**	**Huambo**	Temperature annual range	1.01	1.04, 1.01
		Annual precipitation	1.02	1.02, 1.03
		Precipitation of coldest quarter	0.86	0.80, 0.91
		Soil pH	48.73	6.70, 376.72
		*Non-spatial model: AIC = 452.83; Adjusted r-squared = 0.19*		
	**Uige**	Mean temperature diurnal range	0.93	0.90, 0.95
		Isothermality	1.61	1.45, 1.80
		Precipitation of driest month	0.29	0.22, 0.37
		Precipitation seasonality	1.35	1.25, 1.47
		Precipitation of driest quarter	1.49	1.38, 1.61
		Precipitation of coldest quarter	0.98	0.97, 0.99
		Elevation	1.00	1.00, 1.00
		NDVI	1.00	1.00, 1.00
		Landcover	1.24	1.11, 1.40
		*Non-spatial model: AIC = 773.68; Adjusted r-squared = 0.26*		
	**Zaire**	Temperature seasonality	1.00	1.00, 1.00
		EVI	1.00	1.00, 1.00
		*Non-spatial model: AIC = 74.40; Adjusted r-squared = 0.26*		
		*Spatial model: Residual variance (φ) = 0.002*		
**2021**	**Huambo**	Annual precipitation	1.01	1.01, 1.02
		Precipitation of wettest quarter	0.99	0.98, 0.99
		Elevation	1.00	1.00, 1.00
		Landcover	1.38	1.21, 1.57
		Soil pH	7.50	2.18, 26.01
		*Non-spatial model: AIC = 607.92; Adjusted r-squared = 0.21*		
	**Uige**	Mean temperature of warmest quarter	0.93	0.91, 0.94
		Precipitation of driest month	2.31	1.89, 2.84
		EVI	1.00	1.00, 1.00
		Soil pH	6.41	1.68, 24.71
		*Non-spatial model: AIC = 411.19; Adjusted r-squared = 0.33*		
	**Zaire**	Mean diurnal range	1.03	1.01, 1.06
		Min temperature of coldest month	1.10	1.05, 1.15
		Precipitation of driest month	0.20	0.12, 0.32
		NDVI	1.00	1.00, 1.00
		*Non-spatial model: AIC = 688.05; Adjusted r-squared = 0.37*		
		*Spatial model: Residual variance (φ) = 0.02*		

Odds ratio and 95%CI derived from non-spatial multivariable regression analysis. Metrics of model performance provided for non-spatial regression models and spatial regression models when used. Isothermality = (mean diurnal temperature range/ temperature annual range) x 100. AIC = Akaike information criterion. CI = confidence interval. EVI = enhanced vegetation index. NDVI = normalized difference vegetation index.

## Discussion

This is the first sub-national spatiotemporal analysis of schistosomiasis and STH prevalence in Angola, which depicts the changing predicted risks of these NTDs across Huambo, Uige and Zaire provinces in the context of a school-based PC program. The results of the study constitute spatial decision support tools that not only allow a more detailed understanding of the impact of the school-based PC program but also informs a more targeted approach to ongoing control strategies for both schistosomiasis and STH prevalence in the geographical areas of concern. Of note, is the expanding area of high predicted STH risk throughout Uige and the evolution of co-endemic hotspots for schistosomiasis and STHs in Zaire. This is despite the regular distribution of albendazole for STHs from 2013 and praziquantel for schistosomiasis since 2014 [13]. For Huambo, there has been a general reduction in predicted schistosomiasis risk across the province, becoming largely low risk for schistosomiasis; as well as a reduction in the STH hotspots in the central part of the province.

Control programs for schistosomiasis and STHs are typically operationalized through administrative units [3]; in Angola, the schistosomiasis and STH control program was implemented at the municipality level [13,15]. However, these administrative levels do not necessarily represent a uniform risk for NTDs, such as schistosomiasis and STHs, as demonstrated in this analysis and those in other settings [25–28]. Furthermore, the evaluation of the impact of this control program was performed at the province-level, incorporating enough data to inform decisions with sufficient power, and demonstrated a limited impact in reducing schistosomiasis or STHs across each of the provinces [15]. This analysis provides the necessary complimentary modelling data identifying schistosomiasis and STH hotspots across Uige and Zaire that not only warrants escalation in the frequency of PC delivery to school-aged children, but also consideration for more detailed community-based prevalence surveys in broader populations to better evaluate drivers of transmission and expansion of the school PC program to community-wide mass drug administration (MDA) [24,29]. The expansion of prevalence surveys and drug administration programs requires careful consideration for resource utilization and operational feasibility, particularly in low- and middle-income countries, such as Angola, that are endemic for NTDs. Using these models, the upscaling of control interventions and monitoring activities can be informed at sub-municipality administrative levels, such as communes, that would allow effective concentration of resources to tackle endemic schistosomiasis and STHs, particularly across Uige and north-eastern Zaire, which borders Uige.

The strength of this analysis includes the detailed parasitological, environmental and climatic data used for the geostatistical modelling. However, limitations in our analysis include the use of imperfect field diagnostic tools for which the diagnostic performance was not accounted for in the modelling. For STHs, there is concern for the low sensitivity of Kato-Katz, particularly in settings with low prevalence and light intensity infection [30], as would be expected following PC programs, that could lead to an underappreciation of true STH prevalence derived from our predictive models. For schistosomiasis, the CCA RDT is considered more sensitive than Kato-Katz, particularly in light intensity and egg-negative infections [31], making it a useful diagnostic tool for the predictive models. The detection of haematuria (using Hemastix) as a proxy for *S. haematobium* has demonstrated variable sensitivity but high specificity compared to urine filtration [32,33]. This is likely to lead to conservative estimates for schistosomiasis risk. The use of data exclusively collected in school-age children restricts the representativeness and generalizability of the predicted epidemiology of schistosomiasis and STHs in the broader population. However, our models can not only be used to guide school-based interventions but also community based epidemiological studies. There are also areas with limited empiric parasitological data, although the survey designs attempted to mitigate this by having representation from each municipality across the provinces. An additional important consideration is the influence of the spatial scale when performing geostatistical model-based risk estimates, which can lead to either an under- or over-estimation of disease prevalence and subsequently drug procurement needs, particularly in settings with relatively homogenous disease prevalence [4].

In conclusion, this analysis provides a clear indication as to the concerning trend for expanding areas of high STH infection risk throughout Uige province and the evolution of co-endemic schistosomiasis and STH infection hotspots in Zaire. Meanwhile, there have been slight reductions in higher-risk areas for Huambo for both schistosomiasis and STH infections. These higher risk areas in Uige and Zaire should be prioritized for control with tailored decisions for future PC delivery.

## Supporting information

S1 Material Semivariograms for the non-spatial regression models for schistosomiasis across (a) Huambo, (b) Uige and (c) Zaire provinces in (i) 2014 and (ii) 2021. (DOCX)

S2 Material Regression model coefficients for variables included in the final risk prediction models for schistosomiasis in 2014 and 2021. (DOCX)

S3 Material Semivariograms for the non-spatial regression models for soli-transmitted helminths across (a) Huambo, (b) Uige and (c) Zaire provinces in (i) 2014 and (ii) 2021. (DOCX)

S4 Material Regression model coefficients for variables included in the final risk prediction models for soil-transmitted helminths in 2014 and 2021. (DOCX)
