# Supplementary material for: Spatiotemporal analysis of schistosomiasis and soil-transmitted helminth distribution in three highly endemic provinces in Angola
Source: PLoS Negl Trop Dis. 2025 Apr 8;19(4):e0012974. doi: 10.1371/journal.pntd.0012974 (PMC12013881; doi:10.1371/journal.pntd.0012974)
Supplement: S2 Material — (DOCX) [file pntd.0012974.s002.docx]

**S2 Material.** Regression model coefficients for variables included in the final risk prediction models for schistosomiasis in 2014 and 2021.

| **Year** | **Province** | **Variable** | **Coefficient estimate** | **Standard error** |
| --- | --- | --- | --- | --- |
| **2014** | **Huambo** | Isothermality | 0.012 | 0.017 |
|  |  | Max temperature of warmest month | 0.005 | 0.005 |
|  |  | Annual precipitation | -0.001 | 0.0006 |
|  |  | Precipitation of coldest quarter | 0.005 | 0.007 |
|  | **Uige** | Mean temperature diurnal range | 0.003 | 0.003 |
|  |  | Temperature seasonality | 0.0004 | 0.0002 |
|  |  | Precipitation of coldest quarter | 0.005 | 0.002 |
|  |  | Elevation | -0.0003 | 0.0001 |
|  |  | NDVI | -0.0001 | 0.00003 |
|  |  | EVI | 0.0001 | 0.00005 |
|  |  | Distance to water bodies | 0.000002 | 0.0000006 |
|  | **Zaire** | Precipitation of wettest month | 0.024 | 0.003 |
|  |  | Precipitation of driest month | 1.948 | 0.328 |
|  |  | Distance to water bodies | -0.000006 | 0.000002 |
| **2021** | **Huambo** | Mean annual temperature | 0.027 | 0.006 |
|  |  | Precipitation seasonality | -0.051 | 0.017 |
|  | **Uige** | Mean annual diurnal range | -0.002 | 0.001 |
|  |  | Temperature seasonality | -0.00009 | 0.00009 |
|  |  | Precipitation of wettest month | -0.0002 | 0.0004 |
|  |  | Elevation | 0.0002 | 0.00006 |
|  |  | Distance to water bodies | 0.0000003 | 0.0000002 |
|  | **Zaire** | Isothermality | -0.009 | 0.022 |
|  |  | Temperature seasonality | -0.0003 | 0.0005 |
|  |  | Precipitation of driest month | 0.234 | 0.110 |
|  |  | Precipitation seasonality | -0.008 | 0.010 |
|  |  | EVI | 0.00001 | 0.00004 |
|  |  | Landcover | 0.0003 | 0.030 |

Isothermality = (mean diurnal temperature range / temperature annual range) x 100. EVI = enhanced vegetation index. NDVI = normalized difference vegetation index.
