# Supplementary material for: Spatiotemporal analysis of schistosomiasis and soil-transmitted helminth distribution in three highly endemic provinces in Angola
Source: PLoS Negl Trop Dis. 2025 Apr 8;19(4):e0012974. doi: 10.1371/journal.pntd.0012974 (PMC12013881; doi:10.1371/journal.pntd.0012974)
Supplement: S3 Material — (DOCX) [file pntd.0012974.s003.docx]

**S3 Material.** Semivariograms for the non-spatial regression models for soil-transmitted helminths across (A) Huambo, (B) Uige and (C) Zaire provinces in (i) 2014 and (ii) 2021.
