# Supplementary material for: Spatiotemporal analysis of schistosomiasis and soil-transmitted helminth distribution in three highly endemic provinces in Angola
Source: PLoS Negl Trop Dis. 2025 Apr 8;19(4):e0012974. doi: 10.1371/journal.pntd.0012974 (PMC12013881; doi:10.1371/journal.pntd.0012974)
Supplement: S4 Material — (DOCX) [file pntd.0012974.s004.docx]

**S4 Material.** Regression model coefficients for variables included in the final risk prediction models for soil-transmitted helminths in 2014 and 2021.

| **Year** | **Province** | **Variable** | **Coefficient estimate** | **Standard error** |
| --- | --- | --- | --- | --- |
| **2014** | **Huambo** | Isothermality | 0.060 | 0.035 |
|  |  | Max temperature of warmest month | -0.007 | 0.015 |
|  |  | Annual temperature range | 0.006 | 0.005 |
|  |  | Precipitation seasonality | 0.002 | 0.002 |
|  |  | Precipitation of coldest quarter | 0.004 | 0.01 |
|  |  | Elevation | -0.0001 | 0.0005 |
|  |  | NDVI | 0.00003 | 0.00005 |
|  |  | Soil pH | 0.305 | 0.412 |
|  | **Uige** | Mean diurnal range | -0.014 | 0.008 |
|  |  | Temperature seasonality | -0.006 | 0.0009 |
|  |  | Annual precipitation | 0.001 | 0.0005 |
|  |  | Precipitation of driest month | -0.885 | 0.164 |
|  |  | Precipitation seasonality | 0.272 | 0.042 |
|  |  | Precipitation of driest quarter | 0.314 | 0.033 |
|  |  | Precipitation of coldest quarter | -0.039 | 0.006 |
|  |  | NDVI | 0.0005 | 0.00008 |
|  |  | Landcover | 0.289 | 0.059 |
|  | **Zaire** | Temperature seasonality | 0.0001 | 0.0003 |
|  |  | Annual temperature range | -0.006 | 0.012 |
|  |  | Precipitation seasonality | -0.004 | 0.012 |
|  |  | EVI | 0.00004 | 0.00004 |
| **2021** | **Huambo** | Annual precipitation | 0.010 | 0.002 |
|  |  | Precipitation of wettest quarter | -0.014 | 0.003 |
|  |  | Elevation | 0.001 | 0.0005 |
|  |  | Landcover | 0.320 | 0.066 |
|  |  | Soil pH | 2.015 | 0.632 |
|  | **Uige** | Mean temperature of warmest quarter | -0.075 | 0.008 |
|  |  | Precipitation of driest month | 0.838 | 0.103 |
|  |  | EVI | 0.001 | 0.0002 |
|  |  | Soil pH | 1.857 | 0.686 |
|  | **Zaire** | Mean diurnal range | -0.011 | 0.007 |
|  |  | Annual temperature range | 0.009 | 0.013 |
|  |  | Precipitation of driest month | -0.246 | 0.109 |
|  |  | NDVI | 0.00006 | 0.00003 |

Isothermality = (mean diurnal temperature range / temperature annual range) x 100. EVI = enhanced vegetation index. NDVI = normalized difference vegetation index.
